# Blood Melatonin in Breast Milk-Fed Preterm Infants: Longitudinal Biomonitoring to 38 Weeks’ Postmenstrual Age (ProMote Study)

**DOI:** 10.3390/children12111490

**Published:** 2025-11-04

**Authors:** Theano Kokkinaki, Manolis Tzatzarakis, Elena Vakonaki, Nicole Anagnostatou, Theano Roumeliotaki, Eleftherios Panteris, Maria Markodimitraki, Ioanna Kakatsaki, Haridimos Kondylakis, Aristidis Tsatsakis, Eleftheria Hatzidaki

**Affiliations:** 1 Department of Psychology, University of Crete, 74150 Rethymno, Greece; 2 Laboratory of Toxicology, Medical School, University of Crete, 70013 Heraklion, Greeceevakonaki@gmail.com (E.V.); 3 Department of Neonatology/Neonatal Intensive Care Unit, University Hospital of Heraklion, School of Medicine, University of Crete, 70013 Heraklion, Greece; nicolehilda@gmail.com (N.A.); eleftherios.panteris@gmail.com (E.P.); medp2012176@med.uoc.gr (I.K.); el.hatzidaki@uoc.gr (E.H.); 4 Department of Social Medicine, School of Medicine, University of Crete, 70013 Heraklion, Greece; roumeliot@uoc.gr; 5 Department of Preschool Education, University of Crete, 74150 Rethymno, Greece; markodim@uoc.gr; 6 Computational Biomedicine Laboratory, FORTH-ICS & Computer Science Department, University of Crete, 70013 Heraklion, Greece; 7 Center of Toxicology & Science Applications, Medical School, University of Crete, 70013 Heraklion, Greece; tsatsaka@uoc.gr; 8 Universidad Ecotec, Km. 13.5 Samborondon, Samborondon 092302, Ecuador; 9 Sechenov IM, First State Medical University, 119991 Moscow, Russia

**Keywords:** preterm infants, melatonin, postmenstrual age, weight-for-gestational-age, linear mixed-effects models, breastfeeding, NICU

## Abstract

**Background/Objectives:** Melatonin, produced by the placenta and pineal gland, regulates circadian timing and has antioxidant and immunomodulatory actions. After birth, neonatal secretion is low and its circadian pattern matures over months; evidence in preterm neonates is mixed. We longitudinally monitored morning blood melatonin from birth to 38 weeks’ postmenstrual age (PMA) in breast milk-fed preterm neonates, assessing differences by time of birth (day vs. night), PMA, and weight-for-gestational-age (WfGA). **Methods:** A prospective NICU cohort, conducted within the ProMote study. In total, 132 preterm neonates were recruited from 112 mothers. For infants ≥33 weeks’ GA, three samples were obtained: umbilical cord (available in 94; otherwise at the first NICU admission), day of life (DOL) 4–7, and DOL 10–14; for infants <33 weeks’ GA, an additional sample at 35–36 weeks’ PMA. Melatonin was measured by ELISA. Primary analyses used raw melatonin concentrations in linear mixed-effects models; sensitivity analyses checked robustness. **Results:** A final sample comprised 122 neonates. Concentrations were low to modest with wide between-neonate variation and no monotonic change across PMA. Mixed models showed no consistent differences by time of birth and no stable WfGA effect; occasional PMA-specific contrasts did not recur at adjacent time points. Umbilical cord concentrations were low, and gestational age at birth did not materially influence levels at a given PMA. Sensitivity analyses led to the same inference. **Conclusions:** In breast milk-fed preterm neonates, morning serum melatonin from 26–38 weeks’ PMA shows substantial individual variability without consistent differences by time of birth, PMA, or WfGA.

## 1. Introduction

Melatonin (MLT) is a neurohormone synthesized primarily by the pineal gland and, to a lesser extent, by extra-pineal tissues such as the placenta. Beyond circadian timekeeping, MLT exerts antioxidant, anti-inflammatory, anti-apoptotic, and immunomodulatory actions and interacts with the gut microbiota [1,2,3,4].

Throughout gestation, melatonin is an essential hormone with antioxidant, anti-inflammatory, and circadian regulatory roles that help maintain immune homeostasis, placental integrity and a stable intrauterine environment for mother and fetus [1,5,6]. During pregnancy, the placenta synthesizes melatonin and is a major contributor to maternal circulating levels [1,7]. Melatonin crosses the placenta and the blood–brain barrier supporting fetal growth [5,6,7,8]. Endogenous melatonin production is minimal in the neonatal period. During the first 3 months, approximately, the infant will experience a transient deficiency in melatonin, due to suboptimal melatonin production and immature circadian rhythmicity [9]. This property has special importance in preterm newborns, since they are not exposed to the final stage of pregnancy when maternal melatonin supply to the fetus is the highest. Thus, in preterm neonates, the early transient period of melatonin deprivation is even more prolonged, making them more vulnerable to chronobiotic dysregulation and inflammatory conditions [4,6,8]. Breast milk is the principal physiologic source of melatonin early in life [10,11]. Human milk carries a robust circadian signal that mirrors maternal secretion [3,10,11,12].

Limited evidence shows that preterm neonate plasma or urine melatonin concentrations are correlated with gestational age, that is, preterm of low GA, at a cut-off of 34 weeks, have significantly lower MLT concentrations compared to preterms of later GA [13], or MLT and MLTS are higher in the more preterm infants (26–32 weeks) compared to the less preterm infants (33–42 weeks) [14]. Alternatively, there is no significant correlation between gestational age and the amount of MLT excretion in 24 h [14]. Regarding changes in neonate plasma MLT concentrations in the early postpartum period, limited evidence shows no increase [13,15] or the amount of MLT increased [14,16]. Also, there is scarce and inconsistent evidence on the rhythm of melatonin levels of preterm neonate umbilical cord, urine MLT excretion, or umbilical artery and venous blood, showing either no significant day/night differences, or a rhythm of melatonin secretion [14,17]. These variations may be attributed to methodological differences among the relevant studies, which investigated neonate MLT in relation to a limited range of PMA (at birth and days 1–8) while only one study measured MLT at days 10, 25, and 55 [13]. What is more, to the best of our knowledge, no previous study has investigated preterm serum melatonin levels of a wide range of gestation age according to weight-for-gestational age. To address these methodological gaps, this study aims to achieve the following:

(i) Examining daytime serum melatonin concentrations in preterm neonates according to repeated measurements between 26 and 38 weeks postmenstrual age (PMA) and according to weight-for-gestational-age (WfGA), as calculated by Fenton growth curves [18,19].
(ii) Quantifying and bounding differences in melatonin of the umbilical cord blood by time of birth (day vs. night).

## 2. Materials and Methods

### 2.1. Study Population

The ProMote study is an ongoing longitudinal study of mothers and their premature neonates conducted at the Department of Neonatology/Neonatal Intensive Care Unit of the University General Hospital of Heraklion. Collection of the data presented here lasted from 10 November 2023 to 31 March 2025. The study protocol [20] has been approved by the Research Ethics Committee of the University of Crete (103/22 September 2023, 158/15 December 2023, and 38/15 February 2024) and by the Scientific Council, according to the positive recommendation of the Ethics Committee, and the Board of Directors of the General University Hospital of Heraklion (26636/2 October 2023 and 35546/23 October 2023). This study complies with the Declaration of Helsinki. Eligibility required maternal intention to feed the infant with mother’s own milk exclusively and to continue breastfeeding for at least the first 28 days of life. It is noted that the Department of Neonatology/Neonatal Intensive Care Unit of the University General Hospital of Heraklion does not have a human milk bank. Mothers were informed by a researcher and received a written information sheet; adequate time was allowed for consideration, after which written informed consent was obtained for both mother and neonate. In total, 132 preterm neonates were recruited from 112 mothers and stratified by gestational age into three groups: very preterm (<32 weeks), moderate preterm (32 0/7–33 6/7 weeks), and late preterm (34 0/7–36 6/7 weeks). The NICU received natural daylight during daytime hours, providing clear daytime cues. At night, light exposure was minimal: incubator covers were used routinely, and room lights were switched on only when clinically necessary and for short periods. Incubator covers were also applied during very bright daytime periods, which are common in Mediterranean settings such as Crete, Greece. Mothers’ own milk was administered as individual doses. Feeds were not time-stamped.

### 2.2. Data Collection

Sociodemographic and maternal data were collected via interview with the mother and included: maternal age, civil status, educational level, employment situation, smoking habits, housing conditions, medical history; including obstetric adverse outcomes (e.g., preeclampsia, gestational diabetes), assisted reproduction techniques and dietary intake during pregnancy. Neonatal data were collected from medical records and included: sex, gestational age and anthropometric measurements (weight, height, head circumference) at birth, oxygen therapy and endotracheal intubation.

### 2.3. Blood Melatonin Collection Procedure

At each preterm delivery, an umbilical cord serum sample (1–2 mL) was collected to determine the baseline melatonin concentration at birth. For infants born at ≥33 weeks gestational age (GA), three samples were obtained: umbilical cord blood—or, if unavailable, the first blood sample drawn on NICU admission—then on day of life (DOL) 4–7, and on DOL 10–14. For infants born at <33 weeks’ GA, four samples were obtained: the same three time points plus an additional sample when the infant reached 35–36 weeks’ postmenstrual age (PMA).

Blood serum was collected by the clinic’s medical staff during scheduled blood test routines, so that no extra interventions were performed on the neonates for the purpose of this study. All neonate blood samples were drawn between 8:00 and 10:00 a.m., and the mean quantity was 250 μL. All samples were centrifuged at 3000× *g* for 5 min, and then the serum was separated and frozen at −80 °C until analysis. The samples were extracted in accordance with the manufacturer’s instructions (ELISA kit, TECAN, IBL International GmbH, Hamburg, Germany; Cat. No. RE54021). The samples were thawed and then were subjected to solid-phase extraction (SPE) with C18 columns. During the procedure, the columns were activated under vacuum with 1 mL of LC-MS grade methanol, followed by 1 mL of distilled water. Of each sample, 250 μL was passed through each column followed by 500 μL of bidistilled water, and two washes with 1 mL of 10% methanol in water, after replacing the glass tube, 1 mL of LC-MS grade methanol was used for the elution of the extract. The samples were dried for 10 min under vacuum and then reconstituted in 150 μL of bidistilled water. The same procedure was used to extract the ELISA kit’s controls and calibrators.

The determination of melatonin levels was performed by enzyme-linked immunosorbent assay (ELISA, IBL International GmbH, RE54021), following the manufacturer’s instructions. All analyses were carried out in duplicate. A volume of 50 μL of each extract was placed into the wells of the Microtiter Plate, followed by the addition of 50 μL of Melatonin Biotin and 50 μL of Melatonin Antiserum. Incubation was performed for 14–20 h at 4 °C. The wells were then washed with the kit’s Wash Buffer, after which 150 μL of freshly prepared Enzyme Conjugate was added to each well and incubated for 120 min at 18–25 °C. The microtiter plate, after the incubation, was washed three times with a wash buffer, and 200 μL of PNPP substrate solution was added to each well, followed by incubation for 40 min at 18–25 °C on an orbital shaker (500 rpm). After incubation, 50 μL of PNPP stop solution was added, and the optical density was immediately measured with a photometer at 405 nm (reference wavelength: 600–650 nm).

With the aid of the calibrators, the standard curve was established, based on which the melatonin concentration in the samples was calculated. Since the sample volume was less than 250 μL, a back-calculation using the corresponding dilution factor for each sample was performed where necessary.

### 2.4. Data Analysis

Melatonin concentrations were summarized using median and interquartile range (IQR) to reflect the non-parametric nature of the distribution. Groups were stratified by birth time according to the timing of peak melatonin levels: day (05:00–16:59) and night (17:00–04:59). Baseline (t_0_) comprised umbilical cord blood when available (N = 75) and, if not, the first peripheral sample obtained within 24 h of life (N = 28), because cord blood is neonatal in origin and acceptable for postnatal testing [21,22]; paired-sample studies show clinical interchangeability for admission testing, thereby minimizing iatrogenic phlebotomy [22,23]. For melatonin specifically, cord blood contains measurable concentrations at delivery, and infants do not exhibit a day–night rhythm during the first postnatal days, supporting comparability between cord and ≤24 h peripheral samples for baseline classification [24,25,26].

Repeated sampling of the t_2_/t_3_ group represents a pooled dataset from later collections, the one closer to 35–36 weeks of postmenstrual age.

Repeated serum melatonin measurements were obtained from preterm infants between 26 and 38 weeks postmenstrual age (PMA). Melatonin concentration was the primary outcome. Owing to right-skewed distribution, analyses were additionally conducted on the natural-logarithmic scale to yield ratio (percent) effects; raw-scale results were reported for comparability with prior literature. Birth time was modelled as day vs. night (TIMEGROUP) and, in sensitivity analyses, as a continuous 24 h rhythm using Fourier harmonics (sine and cosine). All infant samples were drawn between 08:00 and 10:00. day/night refers to time of birth only and is not a circadian phase comparison. Weight-for-gestational-age (WfGA) percentiles and z-scores were calculated from the sex-specific Fenton (2013) preterm growth reference [27]; infants were coded as appropriate-for-GA (AGA), large-for-GA (LGA) and small-for-GA (SGA). Stress-related care (oxygen therapy, endotracheal intubation).

Non-parametric statistical tests were used to examine bivariate correlations, such as Spearman’s correlation coefficient, and group differences, such as Mann–Whitney test.

Multivariate mixed linear regression models with robust variances were used to incorporate the longitudinal nature of the study for assessing the effect of time of birth (day/night groups) and size at birth (AGA vs. SGA or LGA) on preterm neonate melatonin levels from birth to discharge. A priori adjustments were infant sex, multiple pregnancy (twin), maternal age, maternal education (compulsory/secondary/tertiary) and maternal country of origin (Greek/Non-Greek). In addition, melatonin concentration of maternal colostrum was included in the models to explore potential effect on infant blood concentration of melatonin; however, this was not further examined due to major sample reduction and no significant alteration in the findings.

Power calculations were performed for total sample size of at least 100, with a two-sided significance level of 5% and 80% power. To maximize statistical power, melatonin concentration in relation to PMA at sampling was analyzed as a continuous variable, rather than being categorized. To mitigate the risk of model overfitting and unstable estimates of multivariable mixed models in the context of a reserved sample size, we restricted the number of covariates included to those with strong a priori justification and have used model selection criteria, such as AIC/BIC. Stata/SE 13.0 (StataCorp LP, College Station, TX, USA) and IBM SPSS Statistics 26 (IBM Corp., Armonk, NY, USA) were used.

### 2.5. Sensitivity Analysis

Primary models were linear mixed-effects with fixed -level random intercepts, fitted by maximum likelihood (REML = false): (i) birth-time × PMA (continuous), (ii) WfGA × PMA, and (iii) continuous birth time with and without interaction with PMA. Optimization used Nelder–Mead with L-BFGS fallback. From log-scale fits, PMA-specific night/day ratios (30, 34, 36, and 38 weeks) and WfGA ratios (LGA/AGA; SGA/AGA) were derived at typical covariate values. Convergence and the variance–covariance structure were checked. Two-sided *p*-values and 95% confidence intervals are reported, with emphasis on effect sizes and compatibility intervals.

The sensitivity analyses were conducted in Python 3.11.8 with the following package versions: pandas 1.5.3, numpy 1.24.0, statsmodels 0.13.5, patsy 1.0.1, scipy 1.14.1, and openpyxl 3.0.10.

## 3. Results

### 3.1. Baseline Characteristics

A final sample of 122 infants was included due to insufficient volume of samples (N = 10) for melatonin analysis. Most births occurred during the day (05:00–16:59; 76.9%) rather than at night (23.1%), and delivery was predominantly by caesarean section (94.3%). The sex distribution was 51.6% male and 48.4% female. At birth, 68.9% received oxygen therapy, and 8.2% were intubated. Anthropometry at enrolment showed length *mean ± SD* 44.9 ± 3.5 cm (median 45.8 cm, IQR 43.0–47.0), head circumference 31.4 ± 2.0 cm (median 32.0 cm, IQR 30.5–32.5), and weight 2181.9 ± 529.5 g (median 2155 g, IQR 1890–2530). Gestational age averaged 33.6 ± 2.0 weeks (median 34.0, IQR 33.0–35.0), with late preterm infants comprising the majority (57.4%; very preterm 12.3%, moderate preterm 28.7%, extremely preterm 1.6%). By weight-for-gestational-age, AGA predominated (84.4%), with SGA 9.0% and LGA 6.6%. Twin births accounted for 31.2% of the cohort. Maternal age was 34.6 ± 6.7 years (median 34.3, IQR 29.8–39.2); Greek nationality was reported in 90.9% of mothers, and tertiary education was the most common level (58.7%). Table 1 presents baseline demographic and clinical characteristics of the sample. Percentages reflect available data, where occasional missingness was present.

### 3.2. Day Versus Night Births

Table 2 compares infants delivered during the day period (05:00–16:59) with those born at night (17:00–04:59). Day births were modestly but significantly more mature and larger at delivery. Median gestational age was 34.0 weeks (IQR 34.0–35.0) in the day group versus 33.0 weeks (IQR 33.0–35.0) in the night group (*p* = 0.005). Day-born infants were longer (46.0 cm, 46.0–47.0 vs. 43.0 cm, 42.0–45.0; *p* = 0.006), had a greater head circumference (32.0 cm, 32.0–32.5 vs. 31.0 cm, 30.5–32.0; *p* = 0.046), and weighed more at birth (2260 g, 2090–2380 vs. 1985 g, 1850–2150; *p* = 0.031). Maternal age did not differ between groups (34.1 years vs. 34.3 years; *p* = 0.503).

As expected, newborns delivered between 33 and 37 weeks’ gestation were significantly larger at birth than those born before 33 weeks. The median length was 46 cm (IQR 45–48) versus 42 cm (39–43), the head circumference was 32 cm (32–33) versus 29 cm (28–31), and the birthweight was 2335 g (2030–2650) versus 1600 g (1300–1900) (all *p* < 0.001). Maternal age was comparable between groups (34.5 years (29.8–38.9) vs. 34.1 years (29.8–40.1); *p* = 0.632).

### 3.3. Melatonin Analysis

#### Initial Profile of Morning Melatonin by PMA

**Figure 1:**
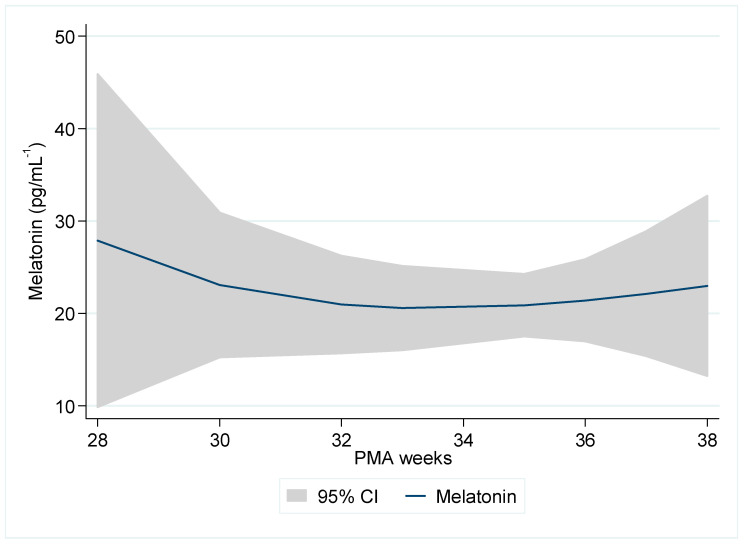
Morning blood melatonin (pg/mL^−1^) by postmenstrual age.

Across 26–38 weeks’ PMA, morning serum melatonin was modest and highly variable (Table 3, Figure 1). Median values clustered around 8–12 pg·mL^−1^ from 31 to 36 weeks (8.2 at 31–32; 9.0 at 34; 11.6 at 35; 10.1 at 36), with wider scatter at some PMAs (SD 27.5 at 35; IQR 30.8 at 36). The 37-week bin showed a higher centre (median 17.8 pg·mL^−1^) but involved few infants (n = 14), and the 38-week bin was very small (n = 6) and lower on average (mean 10.8 pg·mL^−1^; SD 7.7). Overall, there were modest morning concentrations with considerable between-infant variability and no monotonic trend across 26–38 weeks.

Infants delivered during daylight hours (05:00–16:59; n = 93) had a median serum melatonin concentration of 10.71 pg/mL^−1^ (IQR 5.49–31.01) within the first 24 h after birth (t_0_). By the first postnatal week (t_1_, days 4–7), the median rose slightly to 13.11 pg/mL^−1^ (IQR 5.46–31.66), a change that was not statistically significant (*p* = 0.563). However, by the second week (t_2_/t_3_, days 10–14), levels had fallen to 6.73 pg/mL^−1^ (IQR 2.90–15.77), representing a significant reduction relative to the t_1_ value (*p* = 0.027). In contrast, infants born at night (17:00–04:59; n = 28) showed a median melatonin of 11.30 pg/mL^−1^ (IQR 6.24–37.58) at t_0_, followed by a non-significant decline to 8.47 pg/mL^−1^ (IQR 5.09–18.76) at t_1_ (*p* = 0.233). Concentrations at t_2_/t_3_ (11.09 pg/mL^−1^; IQR 3.39–18.63) did not differ from those at t_1_ (*p* = 0.929). Taken together, early postnatal trajectories show small, group-specific fluctuations but no sustained day–night separation in morning samples (Table 4).

### 3.4. Mixed-Models Analysis

**Figure 2:**
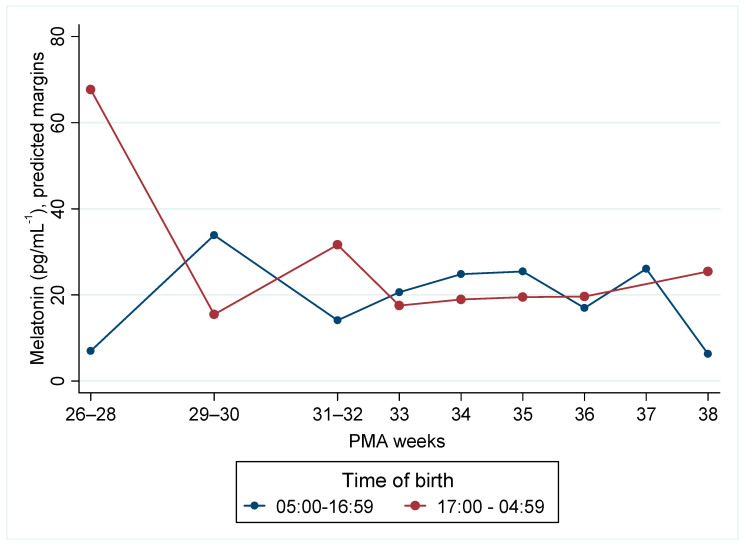
Melatonin by postmenstrual age (PMA) for time-of-birth group (05:00–16:59 vs. 17:00–04:59). Predicted margins were obtained from mixed-effects linear models with random effects for infant and a random slope for PMA, adjusted for sex, multiple pregnancy, maternal age, education, and country of origin.

Serum melatonin was analysed on the raw scale using linear mixed-effects models with a random intercept per infant, adjusting for sex, multiple pregnancy, maternal age, maternal education, and country of origin. In the time-of-birth model (day as reference; PMA modelled continuously with 26–28 weeks as the anchor), there was no overall day–night difference at the reference PMA (*p* = 0.107). Because the model includes a day/night × PMA interaction, the day–night contrast is permitted to vary with maturity. Across PMA, most contrasts were non-significant, with one isolated difference at 29–30 weeks, where night births showed lower melatonin by 79.0 pg/mL (95% CI −154.3 to −3.8; *p* = 0.040). Independent of birth-time group, mean melatonin was modestly higher at 35 weeks relative to 26–28 weeks (+18.5 pg/mL; 95% CI 0.45 to 36.5; *p* = 0.045) (Figure 2).

In the weight-for-gestational-age (WfGA) model (AGA as reference; PMA anchor 31–32 weeks), overall WfGA main effects were not significant (LGA vs. AGA, *p* = 0.262; SGA vs. AGA, *p* = 0.792). A limited number of PMA-specific contrasts did attain significance: LGA exceeded AGA at 37 weeks (+46.5 pg/mL; 95% CI 21.0 to 71.9; *p* < 0.001) and 38 weeks (+21.9 pg/mL; 95% CI 8.0 to 35.8; *p* = 0.002), while SGA was lower than AGA at 34 weeks (−14.3 pg/mL; 95% CI −26.9 to −1.6; *p* = 0.027). Mean melatonin at 38 weeks was also lower than the 31–32-week anchor (−11.8 pg/mL; 95% CI −21.3 to −2.2; *p* = 0.016).

**Figure 3:**
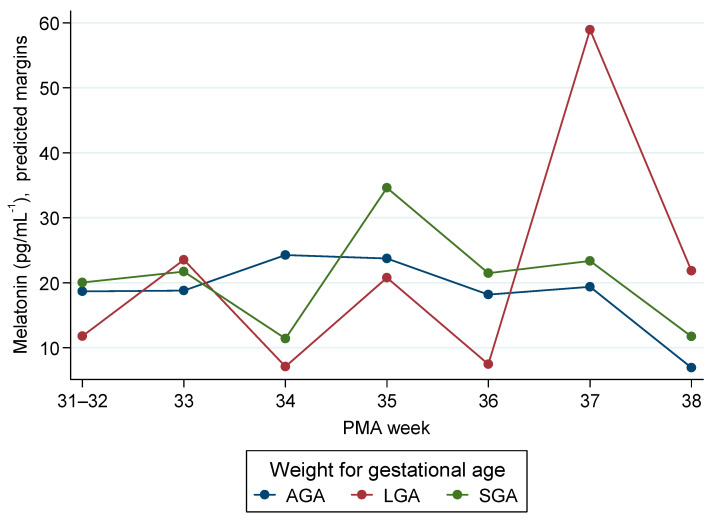
Melatonin by postmenstrual age (PMA; binned) and for the weight-for-gestational-age (WfGA) category (AGA, LGA, SGA). Predicted margins were obtained from mixed-effects linear models with random effects for infant and a random slope for PMA, adjusted for sex, multiple pregnancy, maternal age, education, and country of origin.

These models indicate no consistent separation by birth-time or by WfGA across 26–38 weeks’ PMA. The few significant contrasts arise at specific maturational points—often near the edges of the PMA range where sample sizes are small (e.g., 37–38 weeks)—and sit alongside numerous adjacent non-significant comparisons (Figure 2 and Figure 3).

Gestational age at birth (GA) was incorporated into the primary mixed-effects framework on the raw scale to assess whether infants of the same PMA but differing GA have different melatonin levels. The linear mixed-effects model (random intercept per infant) included PMA and GA (both continuous) with the a priori adjustments (sex, multiple pregnancy, maternal age, education, country), centring PMA at 35 weeks and GA at 33 weeks for interpretation. The overall GA effect at a given PMA was not statistically significant (+1.06 pg/mL per +1-week, 95% CI −1.05 to +3.17; *p* = 0.324), and there was no evidence of a PMA × GA interaction (*p* = 0.246). To align with PMA-specific reporting elsewhere, within-PMA adjusted models (raw scale; cluster-robust SEs by infant) were fitted at 34, 35, and 36 weeks. These were null at 34 weeks (+3.41 pg/mL per week; 95% CI −3.98 to +10.80; *p* = 0.367) and 36 weeks (+0.73 pg/mL; −2.04 to +3.50; *p* = 0.607), whereas at 35 weeks higher GA at birth was associated with higher melatonin (+4.16 pg/mL per +1 week; 95% CI +1.73 to +6.58; *p* = 0.00078), corresponding to an 8.31 pg/mL difference for GA 34 vs. 32 weeks (95% CI +3.46 to +13.16). As prespecified, the log-scale sensitivity analysis was concordant at the aggregate level (no overall GA effect; PMA × GA *p* = 0.56) and showed a parallel PMA-35 signal (per +1 week ratio 1.20, 95% CI 1.09–1.32; GA 34 vs. 32 ratio 1.44, 95% CI 1.18–1.76). Table 5 and Table 6 have the relevant metrics. Full raw-scale and log-scale estimates are provided in Supplementary Tables S4–S7 S-GA.

A sensitivity analysis using log-scale mixed models with the same adjustments was also performed. Given the right-skew of melatonin, we re-specified the outcome on the natural-logarithmic scale with PMA as a continuous term, retaining the same covariate adjustments and random-intercept structure. While residual behaviour improved, the statistical significance of the models did not change. Inferences were unchanged: neither the night main effect nor the night × PMA interaction reached conventional significance, and WfGA × PMA terms were likewise non-significant. For orientation, the adjusted night/day ratios were 1.42 (95% CI 0.76–2.64) at 30 weeks PMA, 1.11 (95% CI 0.78–1.59) at 34 weeks, 0.98 (95% CI 0.63–1.54) at 36 weeks, and 0.87 (95% CI 0.46–1.65) at 38 weeks. Full fixed-effect estimates, and PMA-specific contrasts are provided in the Supplement.

To examine potential influences of care-related stress, we added oxygen therapy and intubation (binary) to the adjusted models, as well as colostrum melatonin concentration in selected models. None showed a clear association with melatonin: oxygen ratio ≈ 0.77 (95% CI 0.54–1.10; *p* = 0.146), intubation ratio ≈ 1.03 (0.59–1.78; *p* = 0.920). There was no evidence of PMA-dependent modification for any of these variables.

## 4. Discussion

This study set out to characterise morning serum melatonin in preterm infants and to ask two questions: (i) whether within-infant changes across subsequent waves (days 4–7, 10–14, and, when applicable, 35–36 weeks’ PMA) differed by birth-time group, postmenstrual age (PMA), or weight-for-gestational-age (WfGA) in raw (unadjusted) mixed-effects models, and (ii) whether time of birth was associated with baseline levels at the first sample (umbilical cord or first NICU draw). At baseline, no birth-time difference was detected. Across repeated measures, trajectories did not separate by birth-time group, PMA showed no monotonic pattern, and WfGA effects were small. Overall WfGA contrasts (LGA/AGA; SGA/AGA) were not significant; PMA-specific LGA elevations at 37–38 weeks occurred in small groups and were not consistent across adjacent PMAs; they are hypothesis-generating rather than confirmatory. Prespecified log-scale sensitivity analyses—summarised as day vs. night and WfGA ratios at PMA 30, 34, 36, and 38 weeks—were concordant and centred near unity. Taken together, these findings suggest that, within this maturation window, any group differences are small relative to between-infant variability.

We showed that across repeated measures, trajectories did not separate by birth-time group. This finding is compatible with what is known about early circadian development. Endogenous melatonin rhythmicity in human infants emerges only gradually in the weeks after birth and may be delayed in those born preterm [11,17,28,29]. Within our 26–38-week PMA window—combined with morning sampling—marked day–night divergence in circulating melatonin would not necessarily be expected. Biological context at delivery is also relevant: cord blood reflects fetal circulation at a time when maternal melatonin still crosses the placenta [24,30], and some umbilical studies report timing or mode-of-delivery effects [31]. After birth, placental input ceases, and differences may attenuate rapidly. Environmental cues in neonatal units further complicate interpretation. Contemporary evidence highlights the role of ambient light in the establishment of infant circadian rhythms and offers practical guidance for neonatal care [32], while reviews of chrononutrition emphasise that melatonin in human milk follows a nocturnal profile that could contribute to entrainment, although interventional data remain limited [8].

Gestational age at birth (GA) was added to the primary mixed-effects framework to test whether infants at the same PMA differ in melatonin by GA. Across 26–38 weeks’ PMA, there was no overall GA effect on the raw scale (+1.06 pg/mL per +1 week, 95% CI −1.05 to +3.17; *p* = 0.324), and no PMA × GA interaction (*p* = 0.246). Within-PMA adjusted models were null at 34 and 36 weeks, whereas at 35 weeks higher GA was associated with higher melatonin (+4.16 pg/mL per week, 95% CI +1.73 to +6.58; corresponding log-scale ratio 1.20, 95% CI 1.09–1.32). Because this signal appears in a single PMA bin, amid multiple comparisons and small cell sizes, it should be regarded as hypothesis-generating rather than confirmatory. If real, slightly higher morning values in LGA infants could reflect an adiposity-related metabolic status, altered hepatic clearance, or residual maternal/placental hormonal influences that modulate neonatal melatonin, but mechanistic interpretation remains speculative with our dataset. The overall pattern accords with existing evidence that endogenous melatonin rhythmicity emerges gradually and may be delayed in preterm infants, with NICU light and other environmental or maternal cues likely attenuating or temporally localising between-group differences in morning samples [8,11,28,30,32].

In practical terms, these results suggest that morning blood melatonin between 26- and 38-weeks’ PMA does not show robust separation by time of birth or WfGA after adjustment.

In interpreting our morning-only serum measurements, it is important to note that endogenous neonatal melatonin is minimal and only consolidates into a circadian rhythm over the first months of life; consequently, breast milk is likely the principal early-life source of melatonin and a vehicle for maternal time cues [10,11]. Daytime light cues were present through natural daylight and routine daytime care. Nighttime light exposure was minimal because incubator covers were used and room lights were switched on only when clinically necessary and for short periods. Covers were also applied during very bright daytime periods, which are common in Mediterranean settings such as Crete, Greece. Mothers’ own milk was administered as individual doses, but feeds were not time-stamped to the time of expression. These practices provided partial environmental cueing and attenuated potential circadian signals via milk. This context may have reduced the likelihood of detecting strong melatonin rhythmicity in morning samples. Our null findings therefore do not argue against circadian biology. Human milk melatonin shows a robust night–day contrast—peaking nocturnally and near-absent by day—so simple, breastfeeding-aligned measures may preserve these signals: dim-light (or lights-off) night feeds; labelling expressed milk and giving “night milk at night” and “day milk by day”; and avoiding day–night pooling when feasible, especially in preterm care [3,8,12]. NICU cycled-lighting policies can complement these strategies, while milk handling warrants attention: preliminary data suggest high-temperature pasteurization reduces milk melatonin, whereas standard Holder pasteurization may have a smaller effect, underscoring the need for careful protocols when expressed or donor milk is used [32,33,34]. Prospective trials that co-manage light and “time-stamped” milk delivery, with sleep and circadian read-outs, are a logical next step albeit not easy to manage in a NICU setting.

### Strengths and Limitations

Strengths include repeated morning sampling across a clinically relevant maturational window (26–38 weeks PMA), prespecified covariate adjustment, and convergence of inferences across complementary modelling frameworks (raw- and log-scale mixed models with alternative correlation structures and PMA parameterisations). The use of PMA-specific contrasts at predefined maturational points further supports clinically interpretable estimates and bounds.

Limitations include the restricted morning-only sampling, which precludes circadian inference and likely attenuates group contrasts, and, while improving internal comparability, underrepresents nocturnal peaks. Daytime light cues were present, whereas nighttime light exposure was minimal and intermittent. Although milk was given as individual doses, feeds were not time-stamped to expression time, limiting alignment of potential circadian milk signals with infant dosing.

Meanwhile it has to be noted that night-sampling could not be carried out because the study protocol [20] predicted that the neonate blood was collected along with other scheduled blood tests, which are carried out only during daytime hours, so that no extra interventions were performed on the neonates for the purpose of this study. (ii) the majority of preterm neonates of our sample were born by cesarean section, and such surgeries are scheduled for daytime hours when possible. Thus, it is important to understand whether such complications could potentially influence melatonin levels given relevant limited evidence [31].

Finally, as a single-centre study with listwise exclusion for missing covariates, some residual confounding and limitations in generalisability cannot be excluded. While future work should incorporate round-the-clock sampling, paired maternal/milk measures, and light quantification [8,11,28,32,35], it should be acknowledged that such protocols are difficult to maintain in routine NICU care, where clinical urgency and staffing constraints often preclude ideal research conditions.

## 5. Conclusions

Across 26–38 weeks’ PMA, morning blood melatonin showed no consistent differences by time of birth or WfGA, and no overall effect of GA at birth when evaluated within the mixed-effects framework. A local signal at PMA 35 weeks suggests higher melatonin with higher GA, but this was not seen at neighboring PMAs and should be regarded as exploratory. Together with concordant log-scale sensitivity analyses, the results imply that any between-group effects in this window are small. Future studies should incorporate 24 h sampling, objective light characterisation, time-stamped feeding with milk assays, and adequate numbers at specific PMAs to test GA effects with precision.

## Figures and Tables

**Table 1:** Baseline demographic and clinical characteristics (N = 122).

Parameter	N (%) or Median (IQR)
Melatonin (pg/mL^−1^)	
t0-Birth	10.4 (5.6–32.4)
t_1_—4th–7th day of life	10.6 (5.2–26.3)
t_2_—10th–14th day of life	9.4 (6.5–26.8)
t_3_—35th–36th week PMA	8.3 (2.9–15.4)
Time of birth	
05:00–16:59	93 (76.9)
17:00–04:59	28 (23.1)
Delivery type	
Vaginal	7 (5.7)
Caesarean section	115 (94.3)
Infant sex	
Male	63 (51.6)
Female	59 (48.4)
Oxygen at birth	
No	38 (31.2)
Yes	84 (68.9)
Intubated	
No	112 (91.8)
Yes	10 (8.2)
Length (cm)	45.8 (43.0–47.0)
Head circumference (cm)	32.0 (30.5–32.5)
Weight (g)	2155 (1890–2530)
Gestational age (weeks)	34.0 (33.0–35.0)
Preterm category	
Extremely preterm	2 (1.6)
Very preterm	15 (12.3)
Moderate preterm	35 (28.7)
Late preterm	70 (57.4)
Weight for GA	
Small for GA (SGA)	11 (9.0)
Appropriate for GA (AGA)	103 (84.4)
Large for GA (LGA)	8 (6.6)
Twins	
No	84 (68.9)
Yes	38 (31.2)
Maternal age (years)	34.3 (29.8–39.2)
Maternal nationality: Greek	
No	11 (9.1)
Yes	110 (90.9)
Maternal education	
Compulsory	7 (5.8)
Secondary	43 (35.5)
Tertiary	71 (58.7)
Melatonin in Colostrum (pg/mL^−1^)	16.3 (7.3–30.6)

**Table 2:** Main baseline characteristics by birth time group.

Variable	Day (05:00–16:59) Median (IQR)	Night (17:00–04:59) Median (IQR)	*p*-Value
GA (weeks)	34 (33–35)	33 (32–34)	0.005
Length (cm)	46 (44–48)	43 (42–46)	0.006
Head circumference (cm)	32 (31–33)	31 (30–32)	0.046
Weight (g)	2260 (1930–2600)	1985 (1780–2285)	0.031
Maternal age (years)	34.1 (29.7–38.6)	34.3 (29.1–39.7)	0.503

**Table 3:** Morning blood melatonin (pg/mL^−1^) by postmenstrual age (PMA).

PMA	N	Median	IQR
26–28	6	7.8	(2.3–31.2)
29–30	14	15.2	(8.1–31.7)
31–32	27	8.2	(5.5–14.6)
33	34	8.7	(5.2–20.9)
34	46	9.0	(5.7–23.8)
35	81	11.6	(5.5–26.1)
36	50	10.1	(5.6–30.8)
37	14	17.8	(3.7–45.4)
38	6	9.2	(4.3–19.1)
Total	278	10.1	(5.4–25.3)

**Table 4:** Birth time melatonin concentration comparisons.

Birth Time Group *	t_0_ (At Birth or 24 h)	t_1_ (4th–7th day)	t_2_/t (10th–14th day)	*p*-Value (Pairs)	*p*-Value (Pairs)
	Median (IQR)	Median (IQR)	Median (IQR)	(t_1_ vs. t_0_)	(t_2_/t_3_ vs. t_1_)
Day (N = 93) (05:00–16:59)	10.71 (5.49–31.01)	13.11 (5.46–31.66)	6.73 (2.90–15.77)	0.563	0.027
Night (N = 28) (17:00–04:59)	11.30 (6.24–37.58)	8.47 (5.09–18.76)	11.09 (3.39–18.63)	0.233	0.929

* 1 missing.

**Table 5:** Mixed-models analysis of melatonin interactions with PMA week at sampling, by time of birth. #: at week.

Parameters	Effect Estimates	95% CI		*p*-Value
**TIME GROUP (Ref. Day)**	60.7	−13.1	134.4	0.107
**PMA (Ref. 26–28 weeks)**				
29–30	26.9	−11.5	65.2	0.170
31–32	7.1	−11.3	25.6	0.448
33	13.7	−6.0	33.3	0.173
34	17.8	−2.2	37.8	0.081
35	18.5	0.4	36.5	0.045
36	10.0	−7.0	27.0	0.249
37	19.1	−3.8	42.0	0.102
38	−0.7	−17.2	15.9	0.937
**TIME GROUP-PMA Interaction**				
2#29–30	−79.0	−154.3	−3.8	0.040
2#31–32	−43.2	−118.2	31.9	0.260
2#33	−63.8	−138.4	10.9	0.094
2#34	−66.5	−140.9	7.9	0.080
2#35	−66.6	−141.5	8.3	0.081
2#36	−58.0	−134.8	18.8	0.139
2#37	N/A	-	-	-
2#38	−41.5	−113.7	30.6	0.259

Effect estimates and corresponding 95% CIs were obtained from mixed-effects linear models with random effects for infant and a random slope for PMA, adjusted for sex, multiple pregnancy, maternal age, education, and country of origin.

**Table 6:** Mixed-models analysis of blood melatonin interactions with PMA week at sampling, by size at birth. #: at week.

Parameters	Effect Estimate	95% CI		*p*-Value
**Weight for GA (Ref. AGA)**				
LGA	−6.9	−19.0	5.2	0.262
SGA	1.4	−8.7	11.5	0.792
**PMA (Ref. 31–32 weeks)**				
33	0.1	−10.9	11.2	0.983
34	5.6	−6.5	17.6	0.363
35	5.0	−4.3	14.4	0.293
36	−0.5	−11.2	10.2	0.928
37	0.7	−16.0	17.4	0.934
38	−11.8	−21.3	−2.2	0.016
**L/SGA-PMA Interaction**				
LGA#33	11.6	−11.3	34.6	0.321
LGA#34	−10.3	−24.7	4.1	0.162
LGA#35	4.0	−17.9	25.8	0.722
LGA#36	−3.8	−18.6	11.0	0.615
LGA#37	46.5	21.0	71.9	0.000
LGA#38	21.9	8.0	35.8	0.002
SGA#33	1.5	−10.8	13.9	0.806
SGA#34	−14.3	−26.9	−1.6	0.027
SGA#35	9.5	−15.8	34.9	0.462
SGA#36	1.9	−16.9	20.7	0.843
SGA#37	2.6	−18.5	23.7	0.809
SGA#38	3.5	−8.8	15.8	0.581

## Data Availability

The dataset is available upon request from the authors due to legal or ethical reasons.

## Supplementary Materials

The following supporting information can be downloaded at: https://www.mdpi.com/article/10.3390/children12111490/s1. Table S1: Main baseline characteristics by gestational week (GW) group. Table S2: Adjusted night/day ratios at selected PMAs (log-scale model). Table S3: Adjusted WfGA ratios at selected PMAs (log-scale model). Table S4: Gestational age at birth (GA) and serum melatonin at a given PMA (raw-scale primary analysis). Table S5: Within-PMA adjusted models (raw scale; cluster-robust SEs by infant). Table S6: Within-PMA adjusted models (raw scale; cluster-robust SEs by infant) Table S7: Within-PMA adjusted models (log scale; cluster-robust SEs by infant).

## Abbreviations

The following abbreviations are used in this manuscript:

MLT	Melatonin
PMA	Postmenstrual age (gestational age at birth + time since birth)
GA	Gestational age
GW	Gestational weeks
WfGA	Weight-for-gestational-age
AGA	Appropriate for gestational age
SGA	Small for gestational age
LGA	Large for gestational age
NICU	Neonatal Intensive Care Unit
DOL	Day of life
ELISA	Enzyme-linked immunosorbent assay
SPE	Solid-phase extraction
PNPP	p-Nitrophenyl phosphate
SCN	Suprachiasmatic nucleus
aMT6s	6-Sulfatoxymelatonin
6-OHMS	6-Hydroxymelatonin sulfate
IQR	Interquartile range
SD	Standard deviation
CI	Confidence interval
SE	Standard error
ML	Maximum likelihood
REML	Restricted maximum likelihood
L-BFGS	Limited-memory Broyden–Fletcher–Goldfarb–Shanno (optimizer)
