# Supplementary material for: Blood Melatonin in Breast Milk-Fed Preterm Infants: Longitudinal Biomonitoring to 38 Weeks’ Postmenstrual Age (ProMote Study)
Source: Children (Basel). 2025 Nov 4;12(11):1490. doi: 10.3390/children12111490 (PMC12651348; doi:10.3390/children12111490)
Supplement: Supplementary file 1 [file children-12-01490-s001.zip › children-3862347-supplementary.pdf]

SUPPLEMENT

Supplementary Tables — Melatonin mixed-model analyses

Supplementary Table S1. Main Baseline Characteristics by Gestational Week (GW) group

Variable	<33 GW Median (IQR)	33–37 GW Median (IQR)	*p*-value
Length (cm)	42 (39–43)	46 (45–48)	< 0.001
Head circumference (cm)	29 (28–31)	32 (32–33)	< 0.001
Weight (g)	1600 (1300–1900)	2335 (2030–2650)	< 0.001
Maternal age (years)	34.1 (29.8–40.1)	34.5 (29.8–38.9)	0.632

Supplementary Table S2. Adjusted Night/Day ratios at selected PMAs (log-scale model).

PMA (weeks)	Night/Day ratio	95% CI ↓	95% CI ↑	p-value
**30.00**	1.42	0.76	2.64	0.267
**34.00**	1.11	0.77	1.59	0.567
**36.00**	0.98	0.63	1.54	0.939
**38.00**	0.87	0.46	1.65	0.668

Supplementary Table S3. Adjusted WfGA ratios at selected PMAs (log-scale model).

Contrast	PMA (weeks)	Ratio	95% CI ↓	95% CI ↑	p-value
**LGA/AGA**	30	0.49	0.12	2.11	0.342
**LGA/AGA**	34	0.91	0.48	1.75	0.783
**LGA/AGA**	36	1.24	0.63	2.45	0.537
**LGA/AGA**	38	1.68	0.59	4.80	0.330
**SGA/AGA**	30	1.08	0.34	3.42	0.897
**SGA/AGA**	34	1.26	0.70	2.27	0.436
**SGA/AGA**	36	1.37	0.76	2.45	0.294
**SGA/AGA**	38	1.48	0.66	3.33	0.346

Supplementary Table S4. Gestational age at birth (GA) and serum melatonin at a given PMA (raw-scale primary analysis)

Primary model: linear mixed-effects on the raw scale with an infant-level random intercept; fixed effects included PMA (continuous), GA at birth (continuous), sex, multiple pregnancy, maternal age, maternal education (Q1.8b) and maternal country (Q1.1). PMA was centred at 35 weeks and GA at 33 weeks for interpretability. Estimation by ML (REML=False); optimiser L-BFGS, with Nelder–Mead fallback if required. Effects are reported as mean differences in pg/mL.

Model / Contrast	Difference (pg/mL)	95% CI (pg/mL)	p-value	Notes
**Mixed model (no interaction): GA per +1 week (at PMA 35)**	1.1	-1.0 to 3.2	0.324	Adjusted; random intercept
**Mixed model (with PMA×GA): GA per +1 week (at PMA 35)**	1.1	-1.0 to 3.2	0.303	PMA×GA p = 0.25
**Predicted contrast at PMA 35: GA 34 vs 32 (2 weeks)**	2.2	-2.0 to 6.4		Derived from interaction model

Supplementary Table S5. Within-PMA adjusted models (raw scale; cluster-robust SEs by infant)

PMA bin	n (obs)	n (infants)	Per +1 wk GA (pg/mL)	95% CI (pg/mL)	p-value	GA 34 vs 32 (pg/mL, 95% CI)
**34**	44	41	4.5	-5.5 to 14.6	0.374	9.1 (-11.0 to 29.1)
**35**	80	70	4.2	1.7 to 6.6	<0.001	8.3 (3.5 to 13.2)
**36**	49	44	0.7	-2.0 to 3.5	0.607	1.5 (-4.1 to 7.0)

Supplementary Table S6. Within-PMA adjusted models (raw scale; cluster-robust SEs by infant)

Primary model: linear mixed-effects on the natural-logarithmic scale with infant-level random intercepts; fixed effects included PMA (continuous), GA at birth (continuous), sex, multiple pregnancy, maternal age, maternal education (Q1.8b) and maternal country (Q1.1). PMA was centred at 35 weeks and GA at 33 weeks for interpretability. Effects are presented as ratios after back-transformation. Estimation by ML (REML=False); optimiser L-BFGS, with Nelder–Mead fallback if required.

Model / Contrast	Ratio	95% CI	p-value	Notes
**Mixed model (no interaction): GA per +1 week (at PMA 35)**	1.05	0.96 to 1.14	0.279	Adjusted; random intercept
**Mixed model (with PMA×GA): GA per +1 week (at PMA 35)**	1.05	0.96 to 1.14	0.272	PMA×GA p = 0.56
**Predicted contrast at PMA 35: GA 34 vs 32 (2 weeks)**	1.10	0.93 to 1.30		Derived from interaction model

Supplementary Table S7. Within-PMA adjusted models (log scale; cluster-robust SEs by infant)

PMA bin	n (obs)	n (infants)	GA per +1 wk (ratio)	95% CI	p-value	GA 34 vs 32 (ratio, 95% CI)
**34**	44	41	1.17	0.83 to 1.65	0.358	1.38 (0.70 to 2.73)
**35**	80	70	1.20	1.09 to 1.32	<0.001	1.44 (1.18 to 1.76)
**36**	49	44	0.97	0.83 to 1.15	0.741	0.95 (0.68 to 1.31)

Abbreviations: GA, gestational age at birth; PMA, post-menstrual age. Ratios >1 indicate higher melatonin with higher GA at birth. Within-PMA models adjusted for the same covariates as the mixed model; inference uses cluster-robust standard errors by infant.
